# Positive association between the neutrophil percentage-to-albumin ratio and constipation: a retrospective cross-sectional study

**DOI:** 10.3389/fmed.2025.1582175

**Published:** 2025-07-08

**Authors:** Yuping Shu, Zuqing Chen, Minyuan Lu, Nai Mi, Yong Wan, Wei Shen, Zewei Sheng, Hongwu Tao, Xuefeng Liu, Yuedong Liu

**Affiliations:** ^1^The Affiliated People’s Hospital of Fujian University of Traditional Chinese Medicine, Fuzhou, China; ^2^Xinjiang Production and Construction Corps Fifth Division Hospital Respiratory Geriatrics Department, Xinjiang, China; ^3^The Proctology Department of Handan Traditional Chinese Medicine Hospital, Handan, China; ^4^Liaoning University of Traditional Chinese Medicine, Second Clinical College, Shenyang, China; ^5^Third Affiliated Hospital, Liaoning University of Traditional Chinese Medicine, Shenyang, China

**Keywords:** NHANES, NPAR, constipation, risk factor, cross-sectional study

## Abstract

**Introduction:**

The neutrophil-to-albumin ratio (NPAR), a novel marker of systemic inflammation, has been utilized to predict outcomes in patients with cancer and cardiovascular diseases. Since inflammation plays a critical role in the pathogenesis of constipation, understanding its connection to NPAR is essential. However, the association between NPAR and constipation remains unclear. This study aims to investigate the potential relationship between NPAR and constipation.

**Method:**

Data from the 2009–2010 National Health and Nutrition Examination Survey (NHANES) were utilized for this study. The neutrophil-to-albumin ratio (NPAR) was calculated as the ratio of the neutrophil percentage to serum albumin levels. To investigate the relationship between NPAR and chronic constipation, various statistical methods were applied, including interaction tests, subgroup analyses, and curve fitting techniques.

**Results:**

Among the 5,011 participants included in the analysis, 366 (7.30%) were identified as having chronic constipation. Higher NPAR levels were significantly associated with an increased likelihood of chronic constipation (OR = 1.05, 95% CI: 1.01–1.10, *p* < 0.05) based on a fully adjusted multiple logistic regression model. Further adjustments revealed that participants in the highest tertile of NPAR had an odds ratio of 1.31 (95% CI: 1.00–1.72, *p* < 0.05) for chronic constipation compared to those in the lowest tertile. Subgroup analyses indicated no significant association in most groups. However, a positive relationship between NPAR and chronic constipation was observed in specific subgroups, including individuals of Other Hispanic ethnicity, smokers, those with heart disease, alcohol consumers, diabetics, and those who were never married.

**Conclusion:**

This study identified a significant positive association between NPAR and the prevalence of chronic constipation. These findings suggest that NPAR may serve as a potential inflammatory biomarker for chronic constipation. Further prospective research is necessary to clarify the long-term implications of elevated NPAR levels on chronic constipation.

## Background

Approximately 10 to 20% of individuals worldwide experience symptoms of constipation. Traditionally, the term “constipation” has been associated with hard stools or irregular bowel movements. However, its clinical presentation varies widely, with symptoms such as anal obstruction, hard stools, straining during defecation, a sensation of incomplete bowel emptying, or the need for physical assistance to facilitate bowel movements (1, 2). Constipation can significantly impact an individual’s social, mental, and physical well-being, yet only 20% of those affected seek medical attention. In the United States, the high prevalence of constipation contributes to approximately 8 million medical visits annually (3) and an estimated $230 million in healthcare costs (4). The etiology of prolonged constipation is multifaceted, involving factors such as intestinal neurotransmitter dysregulation, fluid balance, colonic motility, sensory dysfunction of the colon, anorectal functional abnormalities, and dietary and lifestyle factors. Among these, dietary habits and lifestyle choices are particularly influential in the development of chronic constipation (5). Adjustments in these areas are considered effective, modifiable factors in managing the condition. The causes of constipation can be categorized into secondary and primary (idiopathic) origins. Chronic idiopathic constipation is further divided into functional defecation disorders, slow-transit constipation (STC), and constipation-predominant irritable bowel syndrome (IBS-C). These subtypes are not mutually exclusive and often overlap significantly (6).

The neutrophil-percentage-to-albumin ratio (NPAR) is an emerging biomarker for systemic infection and inflammation. It is calculated by dividing the neutrophil percentage by the serum albumin concentration, combining these two parameters into a single, meaningful indicator (7). Recent studies suggest that NPAR may serve as a prognostic marker for various conditions, including chronic kidney disease, cardiogenic shock, severe sepsis, cancer, nonalcoholic fatty liver disease, and advanced liver fibrosis (8–12).

Despite its potential, limited research has explored the role of NPAR in patients with constipation. To address this gap, we utilized data from the 2009–2010 National Health and Nutrition Examination Survey (NHANES) to conduct a large-scale, cross-sectional analysis of the US civilian population. Our objective was to investigate the potential association between NPAR and constipation, aiming to provide novel insights and practical recommendations for the clinical management and prevention of constipation.

## Method

### Survey description

**Figure 1:**
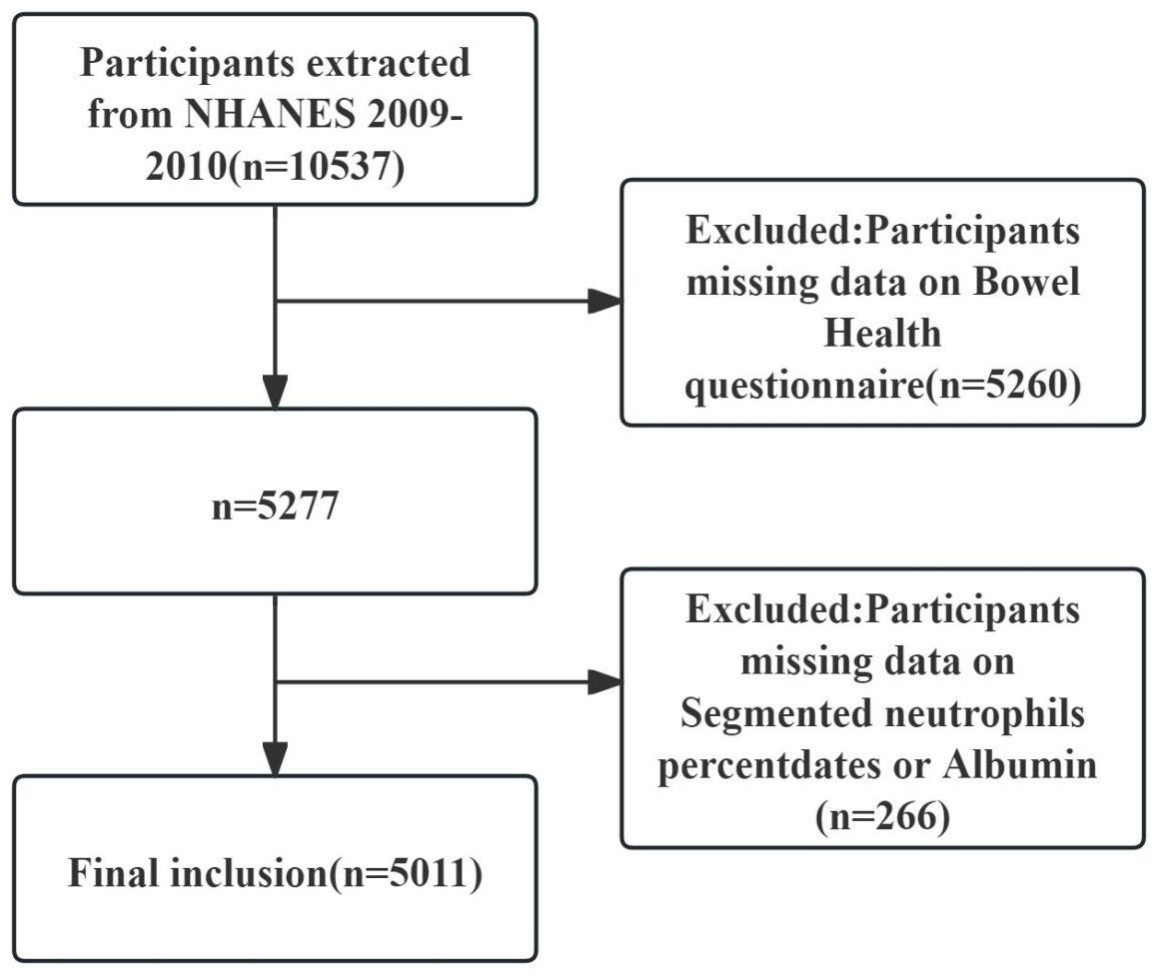
2009–2010 NHANES participant selection flowchart.

The National Health and Nutrition Examination Survey (NHANES) is a comprehensive program conducted in the United States to gather health-related data from a representative sample of the population. Utilizing a cross-sectional study design, NHANES employs stratified, multistage, and random sampling methods to ensure the data reflects the health and nutritional status of the general U. S. population. The survey is carried out by the National Center for Health Statistics (NCHS) under the Centers for Disease Control and Prevention (CDC) (13). Its primary objective is to assess dietary habits, overall health, and the nutritional and health status of both adults and children in the U. S. All participants provided written informed consent, and the study was approved by the Research Ethics Review Board of the NCHS (14). For this investigation, publicly available gastrointestinal health data from the 2009–2010 NHANES dataset were utilized. The initial dataset included 10,537 individuals aged 20 years or older. Participants completed a questionnaire assessing bowel movement frequency and basic stool characteristics. To ensure accuracy and reliability, exclusion criteria were applied: individuals without data from the gastrointestinal health questionnaire (*N* = 5,260) and those missing information on segmented neutrophil percentages or albumin levels (*N* = 266) were excluded. After applying these criteria, the final analysis sample consisted of 5,011 participants. A detailed overview of the participant screening process is provided in Figure 1.

### The definition of constipation

Constipation was characterized using data from the NHANES database, which included information on bowel movement frequency and stool consistency (15). Participants reported the frequency and texture of their stools over the 30 days preceding data collection. Stool consistency was assessed using the Bristol Stool Form Scale (BSFS), a tool featuring visual cards with descriptions and images of seven stool types.

Following established criteria from previous studies (16–19), individuals who identified BSFS type 1 (separate hard lumps, resembling nuts) or BSFS type 2 (sausage-shaped but lumpy) as their typical or most common stool type were classified as having chronic constipation. Additionally, participants who reported using laxatives at least once per week over the previous 30 days were also categorized as chronic constipators. Conversely, individuals who indicated BSFS type 6 (fluffy, ragged-edged pieces with a mushy consistency) or BSFS type 7 (watery, no solid pieces) as their usual stool type were classified as having chronic diarrhea. All other participants, whose stool types did not fall into these categories, were classified as having regular bowel movements.

### Assessment of NPAR

Hematologic parameters were obtained from the NHANES Complete Blood Count (CBC) Profile. These parameters were measured using the Beckman Coulter method, which involves enumerating and sizing blood cells. This process incorporates an automated dilution and mixing system for sample preparation and a single-beam photometer for hemoglobin measurement. The white blood cell (WBC) differential was determined through Volume, Conductivity, and Scatter (VCS) technology.

Albumin levels were measured as part of the NHANES Standard Biochemistry Profile using the LX20 instrument. This system utilizes a bichromatic digital endpoint technique, where albumin binds to Bromocresol Purple (BCP) to form a complex. The absorbance change at 600 nm is measured, with albumin concentration directly proportional to this change. The neutrophil-to-albumin ratio (NPAR) was calculated using data from the same blood samples. The formula used for calculation was as follows:


NPAR=Neutrophil Percentage(%)/Albumin(g/dL)

This approach provides a composite biomarker reflecting both inflammatory activity and nutritional status (7).

### Covariate

To minimize the impact of potential confounding variables on the association between NPAR and health outcomes, the study adjusted for a comprehensive set of covariates. These included socioeconomic status (poverty-to-income ratio, PIR), lifestyle factors (e.g., smoking and alcohol use), demographic variables (age, gender, and race/ethnicity), and clinical parameters (body mass index, pre-existing health conditions, and hypertension) (5, 20). Participants were categorized as smokers if they were current smokers who had smoked at least 100 cigarettes in their lifetime. Non-smokers were defined as individuals who had either never smoked or smoked fewer than 100 cigarettes. Alcohol consumption was assessed by identifying individuals who consumed at least 12 alcoholic beverages during 2009 and 2010, classifying them as drinkers. Diabetes status was determined based on either a glycated hemoglobin (HbA1c) level of ≥6.5% or a self-reported diagnosis of diabetes (21). Hypertension was defined as having a systolic blood pressure ≥140 mmHg, diastolic blood pressure ≥90 mmHg, or current use of antihypertensive medications.

### Statistical analysis

Participants were divided into three groups based on their NPAR levels, and demographic characteristics were analyzed using chi-square tests and t-tests. Weighted multivariable logistic regression models were employed to assess the linear relationship between NPAR and chronic constipation. To further evaluate the association, NPAR was categorized into tertiles, and a trend analysis was performed to examine its linear relationship with the risk of chronic constipation.

Subgroup analyses were conducted to explore the relationship between NPAR and chronic constipation across various factors, including gender, BMI, alcohol consumption, smoking status, and comorbidities such as hypertension and diabetes. Interaction tests were utilized to evaluate the consistency of these associations across subgroups.

Additionally, nonlinear associations between NPAR and chronic constipation were assessed using smoothing curve fitting techniques. All statistical analyses were performed using R software (version 4.1.3) and EmpowerStats (version 2.0). Statistical significance was defined as a two-sided *p*-value of <0.05.

## Results

### Baseline characteristics

Among the 5,011 participants included in the analysis, the mean age was 49.49 years (SD: 17.69), with a gender distribution of 49.77% male and 50.23% female. Constipation was reported by 366 participants, corresponding to an overall prevalence of 7.30%. The mean NPAR for the cohort was 13.88 (SD: 2.68), which was stratified into tertiles as follows: tertile 1 (T1) with an average of 11.10 ± 1.37, tertile 2 (T2) with 13.78 ± 0.62, and tertile 3 (T3) with 16.76 ± 1.78. Participants in the higher NPAR tertiles tended to be older, predominantly female, more likely to be married, and exhibited higher rates of alcohol consumption compared to those in the lowest tertile. Conversely, individuals in higher tertiles showed lower smoking rates and were less likely to have a never-married status. Notably, the prevalence of constipation increased with rising NPAR levels, ranging from 7.07% in T1 to 8.31% in T3 (Table 1).

**Table 1:** Baseline characteristics of participants from NHANES 2009–2010.

Characteristics	T1 (*N* = 1,669)	T2 (*N* = 1,670)	T3 (*N* = 1,672)	*p*-value	*p*-value^*^
NPAR	11.10 ± 1.37	13.78 ± 0.62	16.76 ± 1.78	<0.001	<0.001
Age	46.42 ± 17.18	48.70 ± 17.11	53.33 ± 18.07	<0.001	<0.001
BMI (kg/m^2^)	27.78 ± 5.59	29.05 ± 6.47	30.84 ± 7.72	<0.001	<0.001
Constipation				0.126	–
No	1,551 (92.93%)	1,561 (93.47%)	1,533 (91.69%)		
Yes	118 (7.07%)	109 (6.53%)	139 (8.31%)		
Gender				<0.001	–
Male	932 (55.84%)	815 (48.80%)	747 (44.68%)		
Female	737 (44.16%)	855 (51.20%)	925 (55.32%)		
Race				<0.001	–
Mexican American	288 (17.26%)	340 (20.36%)	292 (17.46%)		
Other Hispanic	192 (11.50%)	169 (10.12%)	156 (9.33%)		
Non-Hispanic White	709 (42.48%)	872 (52.22%)	915 (54.72%)		
Non-Hispanic Black	384 (23.01%)	217 (12.99%)	235 (14.06%)		
Other Race	96 (5.75%)	72 (4.31%)	74 (4.43%)		
Marital status				<0.001	–
Married	841 (50.39%)	910 (54.49%)	872 (52.15%)		
Widowed	96 (5.75%)	131 (7.84%)	185 (11.06%)		
Divorced	170 (10.19%)	177 (10.60%)	203 (12.14%)		
Separated	54 (3.24%)	49 (2.93%)	55 (3.29%)		
Never married	355 (21.27%)	255 (15.27%)	232 (13.88%)		
Living with partner	152 (9.11%)	146 (8.74%)	124 (7.42%)		
Refused	1 (0.06%)	2 (0.12%)	1 (0.06%)		
Smoking status				<0.001	–
No	1,170 (70.10%)	1,048 (62.75%)	979 (58.55%)		
Yes	499 (29.90%)	622 (37.25%)	693 (41.45%)		
Heart disease				<0.001	–
No	1,620 (97.06%)	1,601 (95.87%)	1,577 (94.32%)		
Yes	49 (2.94%)	69 (4.13%)	95 (5.68%)		
Diabetes				<0.001	–
No	1,529 (91.61%)	1,502 (89.94%)	1,397 (83.55%)		
Yes	140 (8.39%)	168 (10.06%)	275 (16.45%)		
Alcohol consumption				0.008	–
Yes	408 (24.45%)	425 (25.45%)	484 (28.95%)		
No	1,261 (75.55%)	1,245 (74.55%)	1,188 (71.05%)		
Hypertension				<0.001	–
No	1,180 (70.70%)	1,095 (65.57%)	974 (58.25%)		
Yes	489 (29.30%)	575 (34.43%)	698 (41.75%)		

BMI, body mass index. In Table 1, the symbol * denotes the *p*-value for the trend test, which is used to assess whether there is a linear trend in the data as the NPAR tertiles increase. Specifically, it indicates the statistical significance of the trend in the baseline characteristics (such as age, BMI, constipation prevalence, etc.) across the three NPAR tertiles (T1, T2, T3). A *p*-value < 0.05 suggests a statistically significant trend, meaning that the observed changes in the characteristics across the tertiles are unlikely to have occurred by chance.

### The relationship NPAR and constipation

Table 2 summarizes the associations between NPAR and constipation prevalence, evaluated both as a continuous variable and by categorizing NPAR into tertiles for better interpretability. In the unadjusted model (Model 1), the logarithmic transformation of NPAR demonstrated a positive but marginally significant association with constipation (OR = 1.04, 95% CI: 1.00–1.08, *p* = 0.0675). This association became statistically significant after adjusting for socioeconomic and demographic factors in Model 2 (OR = 0.97, 95% CI: 0.94–0.99, *p* = 0.0177). In the fully adjusted model (Model 3), which controlled for all potential confounders, the positive relationship between NPAR and constipation remained significant (OR = 1.05, 95% CI: 1.01–1.10, *p* = 0.0112). This result indicates that each unit increase in the logarithm of NPAR was associated with a 5% higher likelihood of constipation. Sensitivity analysis was conducted by categorizing zinc intake into tertiles. In the fully adjusted model, participants in the highest zinc intake tertile exhibited a reduced risk of constipation compared to those in the lowest tertile, though this association did not reach statistical significance (OR = 1.30, 95% CI: 0.99–1.72, *p* = 0.0599). Nevertheless, a clear trend was observed, with higher NPAR levels correlating with increased constipation prevalence (P for trend < 0.01). These findings underscore a consistent positive association between NPAR and the risk of constipation, suggesting that higher NPAR may serve as an indicator of increased constipation susceptibility.

**Table 2:** Association between NPAR and constipation symptoms [OR (95%CI)].

OR (95% CI), *p* value			
Continuous	Model 1	Model 2	Model 3
	1.04 (1.00, 1.08) 0.0675	1.05 (1.01, 1.10) 0.0112	1.05 (1.01, 1.10) 0.0155
Categories			
Tertile1	1.0	1.0	1.0
Tertile2	0.92 (0.70, 1.20) 0.5331	0.95 (0.72, 1.25) 0.7229	0.95 (0.72, 1.26) 0.7280
Tertile3	1.19 (0.92, 1.54) 0.1780	1.31 (1.00, 1.72) 0.0459	1.30 (0.99, 1.72) 0.0599

In the sensitivity analysis, NPAR was transformed from a continuous variable into a categorical one (tertiles). OR stands for odds ratio, while 95% Cl refers to the 95% confidence interval. Model 1: No adjustments were made for covariates. Model 2: Adjustments were made for gender, age, race, BMI and marital status. Model 3: Adjusted for 1gender, age, race, PIR, BMI, alcohol intake, hypertension, Heart disease, diabetes, Smoke status and marital status.

### Subgroup analysis

The association between NPAR and constipation is generally weak across most subgroups, as indicated by odds ratio (OR) values close to 1 and *p*-values that do not reach statistical significance. However, significant associations were observed in specific subgroups, including Other Hispanic individuals (OR = 1.15, 95% CI: 1.01–1.31, *p* = 0.0396), smokers (OR = 1.06, 95% CI: 1.00–1.12, *p* = 0.0405), non-drinkers (OR = 1.06, 95% CI: 1.01–1.11, *p* = 0.0302), never-married individuals (OR = 1.11, 95% CI: 1.01–1.22, *p* = 0.0247), those with heart disease (OR = 1.05, 95% CI: 1.01–1.10, *p* = 0.0203), and individuals with diabetes (OR = 1.06, 95% CI: 1.01–1.11, *p* = 0.0125). Interaction *p*-values did not reveal significant variability in the effect of NPAR across subgroups, suggesting that for most groups, the relationship between NPAR and constipation is either weak or not statistically significant (see Table 3).

**Table 3:** Subgroup analysis.

Variable	OR (95% CI)	*p*-value	P for interaction
Gender			0.8014
Male	1.06 (0.98, 1.14)	0.1405	
Female	1.05 (0.99, 1.10)	0.0810	
Race			0.6859
Mexican American	1.02 (0.93, 1.13)	0.6487	
Other Hispanic	1.15 (1.01, 1.31)	0.0396	
Non-Hispanic White	1.06 (0.99, 1.14)	0.0751	
Non-Hispanic Black	1.03 (0.95, 1.12)	0.4475	
Mexican American	1.05 (0.88, 1.26)	0.5568	
Smoking status			0.6381
Yes	1.06 (1.00, 1.12)	0.0405	
No	1.04 (0.97, 1.11)	0.2633	
Alcohol consumption			0.6138
No	1.04 (0.96, 1.11)	0.3399	
Yes	1.06 (1.01, 1.11)	0.0302	
Marital status			0.4338
Married	1.05 (0.99, 1.11)	0.1372	
Widowed	1.00 (0.87, 1.16)	0.9553	
Divorced	1.01 (0.87, 1.16)	0.9093	
Separated	0.82 (0.61, 1.12)	0.2186	
Never married	1.11 (1.01, 1.22)	0.0247	
Living with partner	1.08 (0.95, 1.23)	0.2578	
Heart disease			0.6291
Yes	1.05 (1.01, 1.10)	0.0203	
No	1.12 (0.86, 1.47)	0.3935	
Diabetes			0.4270
Yes	1.06 (1.01, 1.11)	0.0125	
No	1.01 (0.90, 1.13)	0.9150	
Hypertension			0.7980
Yes	1.05 (1.00, 1.10)	0.0679	
No	1.06 (0.99, 1.14)	0.1146	

Analyses of subgroups that incorporate covariates adjusted for in Model 3. OR, odds ratio; 95% Cl, 95% confidence interval.

### Non-linear relationship between NPAR and constipation

**Figure 2:**
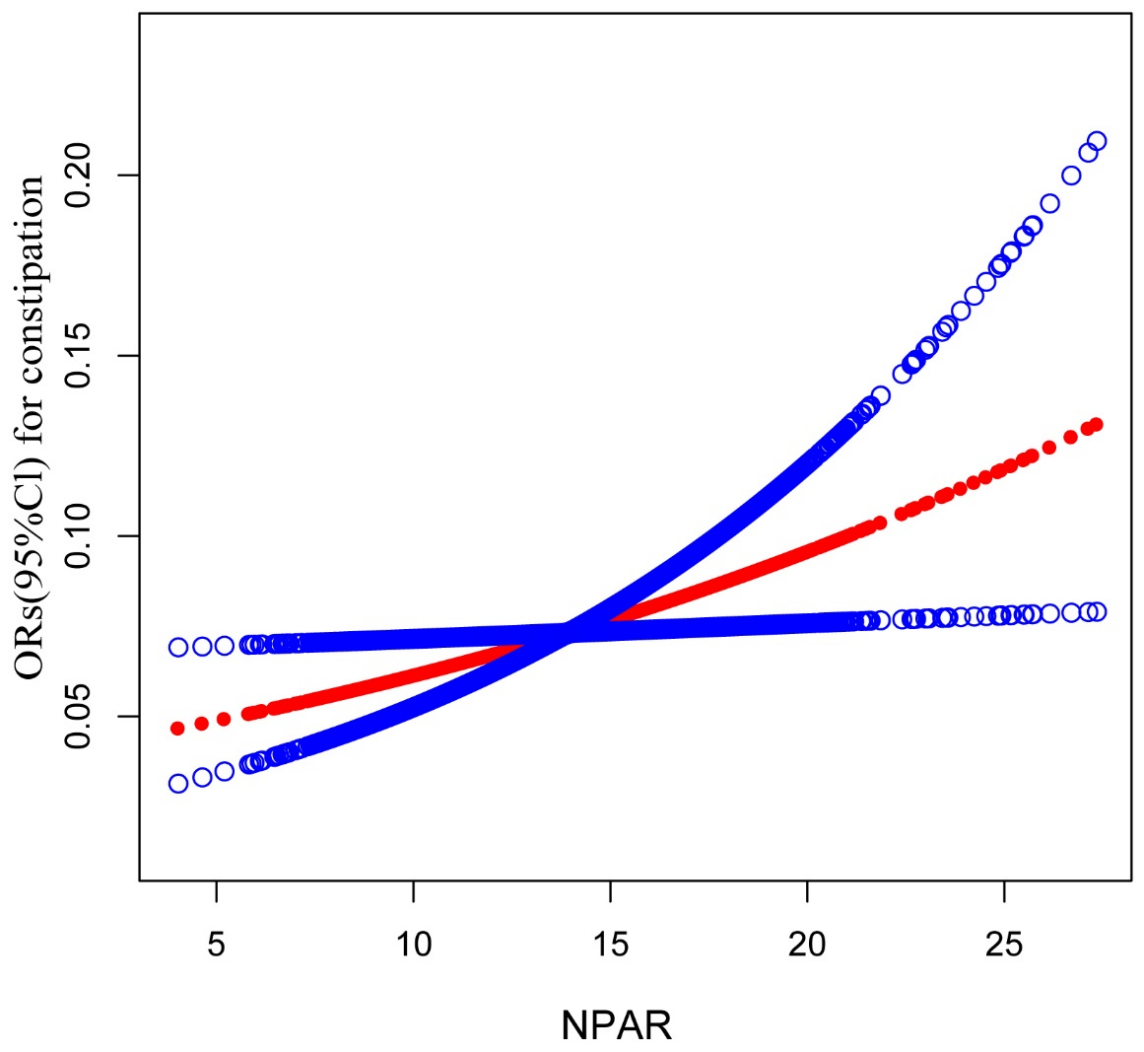
Odds ratios (ORs) with 95% confidence intervals for the association between neutrophil percentage-to-albumin ratio (NPAR) and constipation, demonstrating a positive trend between NPAR and constipation risk.

The relationship between NPAR and constipation was analyzed using a curve-fitting technique, resulting in a graphical representation. The identified positive linear association between NPAR and constipation prevalence is illustrated in Figure 2.

## Discussion

Our cross-sectional analysis of 5,011 participants demonstrated that higher NPAR levels were associated with an increased prevalence of constipation. Subgroup analysis and interaction tests indicated that this association was not significant in most populations. However, a positive correlation was observed in specific subgroups, including Other Hispanic individuals, smokers, non-drinkers, individuals with heart disease, never-married individuals, and those with diabetes.

The prevalence of constipation has been shown to vary across studies, depending on the study design and the criteria used to define constipation (5, 22). For instance, Zhang et al. reported a constipation prevalence of 7.01% based on stool consistency and 3.08% based on defecation frequency (23). In the present study, constipation was assessed using the Bristol Stool Form Scale and data from the Intestinal Health Questionnaire, which considered both stool texture and bowel movement frequency. Consistent with the findings of Liu X. et al. (36) this study identified a constipation prevalence of 7.41% (24, 25).

The neutrophil-to-albumin ratio (NPAR) has been identified as a novel marker for systemic infection and inflammation in humans (11, 26). Elevated NPAR levels result from an increased neutrophil percentage and/or decreased albumin concentrations. Our findings align with previous studies that have highlighted the prognostic value of NPAR in various clinical contexts, including cardiogenic shock (10), acute kidney injury (27), myocardial infarction (28), and gastrointestinal disorders (29). Additionally, research suggests that inflammation plays a critical role in the development and progression of constipation (24, 25).

Constipation is a prevalent and multifaceted gastrointestinal disorder, often attributed to disruptions in gastrointestinal motility (30). Research indicates that a variety of factors contribute to its development. Normal bowel motility depends on several physiological processes, including fermentation, bile acid metabolism, mucus secretion, immune and nervous system functionality, inflammation, and the balance of gut microbiota. Dysregulation or dysfunction in any of these processes can impair bowel movements, resulting in constipation symptoms (31).

Recently, significant attention has been given to the “gut-brain axis” and its role in the interaction between gut microbiota and brain function. Dysbiosis of the gut microbiota has been closely associated with heightened systemic inflammation. Evidence suggests that a compromised intestinal barrier allows harmful bacterial endotoxins, such as lipopolysaccharides (LPS), to enter the bloodstream, triggering systemic inflammatory responses (32). This gut microbiota imbalance is thought to play a pivotal role in the pathogenesis of constipation (33). Our study is the first to establish a direct association between NPAR and constipation, emphasizing the contributions of systemic inflammation and gut microbiota dysregulation to the condition’s development. As a cost-effective and readily accessible biomarker, NPAR shows promise as a risk stratification tool in clinical settings. It could aid in early identification and intervention for individuals at high risk of developing constipation, potentially preventing its onset and facilitating timely treatment. This novel application of NPAR offers clinicians a simple yet effective method for monitoring and managing patients with constipation, particularly those with coexisting inflammatory conditions.

The strengths of our study include the utilization of a large sample size and nationally representative data from the 2009–2010 National Health and Nutrition Examination Survey (NHANES) in the United States. To improve the robustness of our findings, we performed subgroup analyses and adjusted for a range of potential confounders, including social factors that may influence constipation. To the best of our knowledge, this is the first study to explore the association between NPAR and constipation, offering significant clinical and public health insights.

However, several limitations must be acknowledged. First, due to the cross-sectional design of the study, establishing a causal relationship between NPAR and constipation is not feasible. Cross-sectional analyses rely on data collected at a single point in time and do not allow for the evaluation of long-term effects (34). Second, the data used in this study were based on a single blood test. Since blood cells have a relatively short lifespan, repeated measurements over time would likely provide more reliable insights compared to a single test performed at one time point. Additionally, the depletion of albumin and neutrophils is common, which may introduce selection bias (35). Third, while we adjusted for various confounding factors, the influence of unmeasured covariates cannot be entirely excluded. Further prospective studies with larger sample sizes and long-term follow-up are required to elucidate the causal mechanisms underlying the relationship between NPAR and constipation (36).

## Conclusion

In conclusion, our study revealed an independent association between NPAR and the prevalence of chronic constipation, characterized by a non-linear relationship. However, further research is needed to clarify the underlying mechanisms and assess the potential therapeutic value of targeting NPAR in constipation management.

## Data Availability

The original contributions presented in the study are included in the article/supplementary material, further inquiries can be directed to the corresponding authors.
